# An Introduction to Integrative Genomics and Systems Medicine in Cancer

**DOI:** 10.3390/genes9010037

**Published:** 2018-01-12

**Authors:** Xiaolong Cheng, Victor X. Jin

**Affiliations:** Department of Molecular Medicine, The University of Texas Health San Antonio, San Antonio, TX 78229, USA; ChengX3@uthscsa.edu

In this Special Issue (SI), with a theme of “Integrative Genomics and Systems Medicine in Cancer”, we have collected a total of 12 research and review articles from researchers in the field of genomics and systems medicine. Integrative genomics and systems medicine is an emerging field that adopts interdisciplinary approaches to dissect complex diseases, including cancer. For instance, integrative genomics often conducts integrative analyses on high-throughput genomic data with novel computational algorithms and further correlates it with clinical outcomes for the identification of biological pathways and molecular targets for better therapies for cancer patients. Systems medicine, however, dissects the systems of the human body as a whole incorporating biochemical, physiological, and environment interactions and builds predictive and actionable models that understand cancer heterogeneity and complexity. The prevention, diagnosis, and treatment of various types of cancers are clearly the most important tasks and a priority in biomedical research communities. 

Modern molecular biology has been revolutionized with the emergence of high-throughput experimental technologies, such as state-of-the-art genomics (omics) profiling and next-generation sequencing. In parallel, numerous efforts have been made in developing novel algorithmic methods and computational analysis approaches to better interpret the various omics data and further gain biological insights. Two of such recent profiling technologies, Hi-C and ChIA-PET, now allow us to study transcriptional regulation at the three-dimensional (3D) scale. These genomic techniques can not only detect chromatin fragment interactions but also partition the genome into active and repressive chromatin domains as well as high-dimensional topological architecture [1]. To better understand the genome structure at a higher resolution, single-cell level profiling techniques are urgently needed to accurately capture the variability of chromatin domains [2,3]. More and more functionally diversified regulatory elements (REs), including enhancers, silencers, insulators, and boundaries, have been identified to act collaboratively with active promoters via long-range tethering or chromatin looping mechanisms [4], thus the looping paradigm has now been recognized as a basic principle of gene regulation. Meanwhile, a huge amount of sequencing datasets that are generated from the deep sequencing technologies requires us to create specialized databases [5,6] for the accurate curations and annotations, and to facilitate the specific studies. Machine learning has been utilized in mining and modeling biological datasets for quite a while, and has become a powerful tool for helping biologists to interpret the gene lists by comparing them with a database of well-annotated gene sets (e.g., pathways) [7], which is valuable for the gene functional discovery from high-throughput experiments. Meanwhile, statistically deterministic machine learning, such as *K*-means, remain a popular clustering algorithm that nonetheless has useful application to new datasets in cancer. For example, a recent study suggests that there is indeed a clustering substructure present in the underlying cancer genome data, at least for most cancer types [8]. 

At the gene level, a new biophysical model of protein–DNA interactions based on the neighbor dinucleotide dependency model BayesPI2 has been developed [9], where new parameters are restricted to a space defined by the dinucleotide combination of DNA structure properties derived from the DiProDB database [10]. Unlike the machine learning approaches, this model was equipped with interpretable model parameters and the training structure, the inferred shape feature preferences can be further studied in various conditions. Such elegant model allows for us to investigate the dynamical change of DNA shape preferences in different cancer cells, and to further elucidate the finer details of transcription factor–DNA interaction, as well as to predict cancer mutation effects in the future. It is now clear that aberrant expression of microRNA (miRNA or miR) is a critical factor in cancer. Oncogenic genetic alterations are responsible for cancer initiation, gradual enlargement and disorganization of tumor tissues, and, ultimately, metastasis. A key, and perhaps the biggest, challenge in miRNA research is the complexity of the miRNA–mRNA target relationship. By connecting genes sharing common miRNA target sites, a miRNA co-regulation network can be constructed [11], which possesses characteristics of the ubiquitous small-world network. Non-hub genes in the network—those sharing miRNA target sites with small numbers of genes—tend to form small cliques with their neighboring genes, while hub genes exhibit high levels of promiscuousness in their neighboring genes. The network analysis showed that key cancer genes and tumor suppressive miRNAs hold a prominent status in miRNA regulation network. The former possesses higher connectivity in the miRNA co-regulation network and more miRNA binding sites in their 3’-UTRs. The latter has more than expected target genes. This network has the potential to be used to better understand miRNA function and their oncological roles. 

Besides, accumulating evidence suggests that long non-coding RNAs (lncRNAs) or long intergenic non-coding RNAs (lincRNAs) play a significant role in cancer etiology and progression, and may serve as potential diagnostic and prognostic markers for various cancers [12]. By fully utilizing the RNA-seq data, a new research has identified many lincRNAs, both known and novel predicted, from lung adenocarcinoma of never smoker individuals that may play a significant role in cancer development, progression, and patient prognosis [13].

The cancer atavistic theory suggests that carcinogenesis is a reverse evolution process. If true, evolutional information may have implications for improving the accuracy of cancer biomarker selection [14]. Through analyzing the cancer endogenous molecular networks, the study from [15] revealed that the subnetwork originating from Eukaryota could control the unlimited proliferation of cancer cells, and the subnetwork originating from Eumetazoa could recapitulate the other hallmarks of cancer. In addition, investigations based on multiple datasets revealed that cancer driver genes were enriched in genes originating from Eukaryota, Opisthokonta, and Eumetazoa. These results have important implications for enhancing the robustness of cancer prognosis models through selecting the gene signatures by the gene age information. 

In another aspect, the gene regulatory networks (GRNs) of immune cells can indicate cell identity and reveal the dynamic changes of immune cells when comparing their GRNs. By conducting a systematic analysis of the GRNs of key immune cell subsets, a recent work from [16] showed that most of the GRNs of these cells in blood share key important hub regulators, but their subnetworks for controlling cell type-specific receptors are different, suggesting that the transformation between these immune cell subsets could be fast so that they can rapidly respond to environmental cues. By comparing the GRNs of the tumor-infiltrating immune T cells and their corresponding immune cells in blood, it showed that the network size of the tumor-infiltrating immune T cells’ GRNs was reduced when compared to the GRNs of their corresponding immune cells in blood. These results suggest that the shutting down certain cellular activities of the immune cells by cancer cells is one of the key molecular mechanisms for helping cancer cells to escape the defense of the host immune system. These results highlight the possibility of genetic engineering of T cells for turning on the identified subnetworks that have been shut down by cancer cells to combat tumors. Through introducing two new rule-based similarity measures, weighted rank-based Jaccard and Cosine measures, a novel computational framework is proposed to detect Condensed Gene Co-Expression Modules (ConGEMs) through the association rule-based learning system and the weighted similarity scores, which is useful for exploring biomarker modules from transcriptomic data [17]. Through investigating the potential of epigenetics to account for the changes in cancer susceptibility, 12 genes that showed strong predictive values for long-term survival in estrogen receptor positive patients were identified [18]. Importantly, two genes that are associated with improved long term survival, HPSE and RPS9, were identified to be hypomethylated in mammary glands of rats exposed prepuberally to genistein (GEN) or to GEN + Bisphenol A (BPA), respectively, reinforcing the suggested cancer suppressive properties of GEN. For hepatocellular carcinoma (HCC) research, a key problem is to identify when and how the critical transition happens during the HCC initiation period at a molecular level. The work from [19] revealed that low-grade dysplastic nodules (LGDNs) is the tipping point of hepatocarcinogenesis based on a series of gene expression. The survival analysis was further used to validate it as an effective predictor of prognosis for hepatitis C virus (HCV)-induced HCC patients on an independent data. This potential clinical application provides biological insights into the dynamic regulation of the critical transitions during multistep hepatocarcinogenesis.

This SI is designed to present the latest findings about regulatory genomics and systems biology in cancer. We hope that the research articles and reviews that were collected here will broaden our minds in cancer research for both experts in the fields and the young generation of researchers, as well as inspire them to share each other’s ideas.
